# Local Low-Dose Lovastatin Delivery Improves the Bone-Healing Defect Caused by *Nf1* Loss of Function in Osteoblasts

**DOI:** 10.1002/jbmr.42

**Published:** 2010-01-29

**Authors:** Weixi Wang, Jeffry S Nyman, Heather E Moss, Gloria Gutierrez, Gregory R Mundy, Xiangli Yang, Florent Elefteriou

**Affiliations:** 1 Vanderbilt University Medical Center, Department of MedicineNashville, TN, USA; 2 Vanderbilt University Medical Center, Department of Orthopaedics and RehabilitationNashville, TN, USA; 3 Department of Veterans Affairs, Tennessee Valley Healthcare SystemNashville, TN, USA

**Keywords:** neurofibromatosis type I, NF1, fracture, healing, pseudoarthrosis, lovastatin, µCT

## Abstract

Postfracture tibial nonunion (pseudoarthrosis) leads to lifelong disability in patients with neurofibromatosis type I (NF1), a disorder caused by mutations in the *NF1* gene. To determine the contribution of *NF1* in bone healing, we assessed bone healing in the 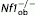 conditional mouse model lacking *Nf1* specifically in osteoblasts. A closed distal tibia fracture protocol and a longitudinal study design were used. During the 21- to 28-day postfracture period, callus volume, as expected, decreased in wild-type but not in 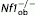 mice, suggesting delayed healing. At these two time points, bone volume (BV/TV) and volumetric bone mineral density (vBMD) measured by 3D micro–computed tomography were decreased in 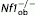 callus-bridging cortices and trabecular compartments compared with wild-type controls. Histomorphometric analyses revealed the presence of cartilaginous remnants, a high amount of osteoid, and increased osteoclast surfaces in 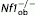 calluses 21 days after fracture, which was accompanied by increased expression of *osteopontin, Rankl*, and *Tgfβ*. Callus strength measured by three-point bending 28 days after fracture was reduced in 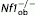 versus wild-type calluses. Importantly, from a clinical point of view, this defect of callus maturation and strength could be ameliorated by local delivery of low-dose lovastatin microparticles, which successfully decreased osteoid volume and cartilaginous remnant number and increased callus BV/TV and strength in mutant mice. These results thus indicate that the dysfunctions caused by loss of *Nf1* in osteoblasts impair callus maturation and weaken callus mechanical properties and suggest that local delivery of low-dose lovastatin may improve bone healing in NF1 patients. © 2010 American Society for Bone and Mineral Research.

## Introduction

Bone fracture healing is a regenerative process that restores bone structural and functional properties through a process reflecting embryonic skeletal development and bone growth. Endochondral bone healing proceeds through the formation of a cartilaginous soft callus that is subsequently replaced by a hard bony callus. Fracture healing is not always successful and can be delayed or not occur at all, leading to severe morbidity. Ninety percent of the nonunion (pseudoarthrosis) cases are associated with neurofibromatosis type I (NF1),(1) a common autosomal dominant disorder caused by mutations in *NF1*.(2–4) *NF1* is a tumor-suppressor gene, and key clinical manifestations of NF1 are neurocutaneous(5–7); however, the skeleton is frequently and variably affected in individuals with NF1.(8) Key skeletal manifestations include decreased bone mineral density (BMD) associated with increased bone resorption, dystrophic or nondystrophic scoliosis, congenital tibia bowing and pseudoarthrosis (nonunion) following fracture.(2,8–12) NF1 long bone bowing typically is associated with the lower distal extremity, usually the tibia,(13) and frequently is followed by fracture, leading to subsequent nonunion, defined as pseudoarthrosis or “false joint.” Treatment options for long bone pseudoarthrosis remain limited, and patients often require multiple surgical attempts for correction and, in some cases, require amputation.(2,14) Such failure in treating this defect underlines the fact that the etiology of NF1 skeletal defects is currently unclear.

Mouse models have been generated to characterize the role of *Nf1* in bone cells. Because *Nf1*^*−*/−^ mice are embryonic lethal,(15) *Nf1*^+/−^ mice have been used. *Nf1*^*+*/−^ osteoprogenitors isolated from *Nf1*^*+*/−^ mice were characterized by increased proliferative activity and impaired differentiation,(16) whereas *Nf1*^*+*/−^ osteoclasts differentiated more efficiently than wild-type (WT) osteoclasts in vitro.(17,18) These defects are accompanied by constitutive activation of RAS and ERK signaling, as observed in other *Nf1*^*+*/−^ lineages.(19) Despite these in vitro observations, *Nf1*^*+*/−^ mice do not display any bone phenotype in vivo under normal conditions. These data and the fact that the NF1 bone-bowing phenotype is congenital, most often unilateral, focal, and not present in every NF1 patient, strongly suggested that *Nf1* loss of function in a subpopulation of mesenchymal cells was responsible for NF1 focal lesions. This hypothesis was supported by identification of *NF1* loss of heterozygosity in bone marrow cells of an NF1 pseudoarthrosis biopsy.(20) Together these observations led us to generate mice lacking both alleles of *Nf1* in osteoblasts. Analysis of this mouse line revealed that lack of *Nf1* in mature osteoblasts impairs osteoblast function and causes a high-bone-turnover phenotype characterized by increased collagen synthesis and bone formation, delayed mineralization, and increased *Rankl*-mediated osteoclastogenesis.(21) Analysis of mice lacking *Nf1* earlier on during the differentiation process in limb mesenchymal osteochondroprogenitors, on the other hand, indicated that *Nf1* deficiency impairs osteoblast differentiation and bone mineralization, causes tibial bowing, and increases bone cortical porosity.(22)

In this study we hypothesized that biallelic loss of *Nf1* specifically in osteoblasts impairs bone healing, and the 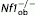 mouse model was used to address this hypothesis. In addition, based on the previously characterized molecular characteristics specific to *Nf1*^−/−^ osteoblasts,(21) which include RAS constitutive activation, we assessed whether the RAS inhibitory properties of lovastatin could ameliorate bone fracture healing in 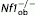 mice using a novel local delivery system that bypasses lovastatin liver metabolism.

## Materials and Methods

### Animals and experimental procedures

All procedures were approved by the Institutional Animal Care and Use Committee at Vanderbilt University Medical Center. WT and 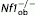 mice were generated by crossing 2.3-kb *α1(I) collagen*-cre *Nf1*^flox/flox^ mice and *Nf1*^flox/flox^ mice, as described previously.(21) All fractures were generated in 2-month-old male mice using the three-point bending method described by Bonnarens and Einhorn.(23) Fifteen mice were assigned per group, and 10 to 12 mice per group were analyzed after eliminating mice with improper fractures (see “Results”). Anesthesia of the animals was induced and maintained with 2% inhaled isoflurane in 100% oxygen. After adequate sedation, the surgical site was sterilized with 70% ethanol, and an incision was made below the right knee. A 27-gauge sterile needle was inserted through the patellar tendon to make an entry point in the tibial epiphysis. Then a 0.25-mm-diameter sterile stainless steel insect pin (Fine Science Tools, Foster City, CA, USA) was inserted into the tibia. Distal tibia fractures were produced using a force exerted by a 220-g weight dropped from a height of 195 mm. Proper fracture position and quality were confirmed via radiography obtained using a digital cabinet X-ray system (LX-60, Faxitron X-Ray, LLC, Wheeling, IL, USA). Mice were allowed to recover on a heated pad and then were placed in recovery cages. Then 0.05 to 0.1 mg/kg of buprenorphine was administered subcutaneously once after the fracture generation and then every 12 hours as required. Lovastatin microparticles (10 mg/kg of mouse body weight) were locally injected adjacent to the fracture site once at the time of fracture. Fracture healing was monitored by weekly radiographs. At 21 and 28 days after fracture, animals were euthanized, and tibias were collected. Surrounding soft tissue and stainless steel pins were removed prior to further analyses. Samples were either frozen in PBS (for biomechanical studies), snap frozen in liquid N_2_ (for molecular studies), or fixed in 10% buffered formalin for 24 hours and then stored in 70% alcohol at 4°C (for µCT and histomorphometric analyses).

### Lovastatin microparticle generation and release profile

Spherical lovastatin microparticles were prepared using an emulsion process. Lovastatin (GMP grade; CNH Technologies, Woburn, MA, USA) was dissolved in reagent-grade methylene chloride. This solution was combined with a chilled aqueous surfactant solution presaturated with methylene chloride using a high-speed mixer. The emulsion so formed was poured into an aqueous extraction bath of chilled water. The organic solvent fully dissolved in the extraction bath, and the insoluble lovastatin particles precipitated. Particles were separated and concentrated by pouring over a series of sieves between 53 and 125 µm in order to achieve the target 70-µm particle size and then rinsed with deionized water. The collected particle suspension then was frozen and lyophilized to yield the free-flowing lovastatin microparticles. Particles were stored at 2 to 8°C until use.

Assessment of in vitro release was performed using 10 mg of lovastatin microspheres. The lovastatin microparticles were placed in a round-bottom 14-mL polypropylene test tube. A 3-mL volume of release medium, 1% polyoxyethylene sorbitan monooleate (Tween 80) in PBS (pH 7.4), then was added. Tubes were capped with serum-separating filters and placed in a water shaker bath at 37°C. At specified times, the supernatant was separated from particles by filtration through the serum filter. All supernatant was removed and a portion retained for analysis. The full supernatant volume was replenished with release medium, the serum filter was replaced, and the tube was returned to the shaker bath. Lovastatin content in supernatant was quantified by HPLC using a stability-indicating method described in *United States Pharmacopoeia*: USP 29.

### Callus evaluation by quantitative µCT

Quantitative characteristics of the callus were evaluated using an ex vivo µCT imaging system (Scanco µCT 40, Scanco Medical, Bassersdorf, Switzerland). Immersed in PBS, the tibia was fit into the specimen tube such that the long axis aligned with the scanning axis. After setting the callus as the region of interest, tomographic images were acquired at 55 kV and 145 mA with an isotropic voxel size of 12 µm and at an integration time of 250 ms with 500 projections collected per 180-degree rotation. For 100 slices above and below the fracture line, contours were fit to the outer perimeter of the callus using the autocontouring feature in the Scanco software. Applying the same threshold to each callus, volume of callus bone tissue (TV), callus bone volume (BV), and volumetric density (in mgHA/cm^3^) of the mineralized tissue (vBMD) were measured as a fraction of mineralized tissue within the callus, excluding the original tibia shaft. Lastly, using a method described by Nyman and colleagues,(24) defects in the outer bridging cortices were quantified, excluding callus trabecular bone and original tibia shaft.

### Structural strength and stiffness by three-point bending assay

Following µCT evaluation, fractured tibias were subjected to biomechanical testing to determine callus structural strength and stiffness. After thawing in PBS, each hydrated tibia was positioned on the bottom two supports (span = 6 mm) such that callus was directly below the top loading point at the end of the actuator (Dynamight 8841, Instron, Canton, OH, USA). An initial preload of 0.5 N was applied to hold the bone in place with the anterior side facing forward. Loading at a rate of 3 mm/min, the data-acquisition software recorded the force from a 100-N load cell and deflection from the linear variable differential transformer (LVDT) at 20 Hz. Callus strength was the maximum force endured by the callus, and callus stiffness was the slope of the linear portion of the resulting force-versus-deflection curve.

### Histologic analyses

Fractured tibias were decalcified in 20% EDTA (pH 7.2) for 1 week at room temperature. Samples then were dehydrated and embedded in paraffin, and 5-µm sagittal sections were cut. Sections were prepared for safranin O/fast green and TRAP staining using standard protocols. Undecalcified samples were processed and embedded in polymethyl methacrylate following standard procedures. Sagittal sections 7 µm thick were obtained through the fracture callus. Sections were stained by the von Kossa/van Gieson methods following standard protocols. Histomorphometric measurements were performed using the Bioquant Analysis System (Nashville, TN, USA).

### Primary osteoblasts extraction, adenovirus infection, and treatment with lovastatin

Calvaria osteoblasts were extracted by multiple collagenase/trypsine digestion from 4-day-old *Nf1*^flox/flox^ pups and cultured in 10% FBS α-MEM. Cells were deprived of serum for 6 hours before infection with adenovirus Ad5-CMV-Cre or Ad5-CMV-eGFP (2500 viral particles per cell; Vector Development Lab, Houston, TX, USA) using 1.2% Gene Jammer transfection reagent (Stratagene, La Jolla, CA, USA), as described by Fouletier-Dilling and colleagues.(25) This strategy allows one to isolate, plate, and multiply cells that are genetically identical (because they are not yet recombined), thus obtaining a homogeneous pool of cells that can be infected with mock (GFP) or cre adenoviruses just prior to the experimental assay (in this case P-ERK measurement). Cells were treated immediately following adenovirus addition with 20 µM lovastatin or vehicle (PBS) for 12 hours before lysis.

### Immunoblotting

Lysates from primary osteoblasts were prepared in RIPA buffer in the presence of protease and phosphatase inhibitors. Proteins were separated by SDS-PAGE and transferred to nitrocellulose membrane using standard protocols. Western blot analyses were performed using anti-total and anti-phospho Erk1/2 (Cell Signaling, Danvers, MA, USA) antibodies, and reaction was detected by chemoluminescence.

### Gene expression assays

Total RNA was extracted using Trizol (Invitrogen, Carlsbad, CA, USA). cDNAs were synthesized following RNase I treatment using the high-capacity cDNA reverse-transcription kit (Applied Biosystems, Carlsbad, CA, USA). Real-time quantitative PCR (RT-qPCR) was performed using TaqMan gene expression assays. The primers for *Rankl* (Mm00441908_m1), *Opn* (Mm00436767_m1), *Tgfβ* (Mm03024053_m1), *Bmp2* (Mm01340178_m1), and the normalizer 18S rRNA were obtained from Applied Biosystems.

### Statistical analysis

Data are expressed as mean ± SEM. Statistical significance was assessed by Student's *t* test. Values were considered statistically significant when *p* < .05.

## Results

### Lack of *Nf1* in osteoblasts delays bone healing

To experimentally address whether *Nf1* in osteoblasts is required for proper bone healing, we compared structural, cellular, molecular, and mechanical parameters of bone healing between WT mice and mice lacking *Nf1* specifically in mature osteoblasts (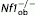 mice).(21) *Nf1*^flox/flox^ mice and 2.3-kb *α1(I) collagen*-cre; *Nf1*^flox/flox^ mice were used as WT and mutant mice, respectively. In an effort to reproduce the typical human NF1 tibial lesion that occurs in pediatric patients and most often at a distal level, we generated stabilized distal tibia fractures in growing mice. At the time of lesion, tibia X-rays were performed to check fracture quality and to eliminate animals with unsatisfactory fractures, namely, comminuted fractures, inaccurate anatomic location, or bent pins. Mice with rotated limbs, as defined by misalignment of the tibia with the tarsus, were excluded. Two days following fracture, animals were able to ambulate and thrive adequately, and no differences in body weight were observed between groups at the endpoint of the study (data not shown).

**Fig. 1:**
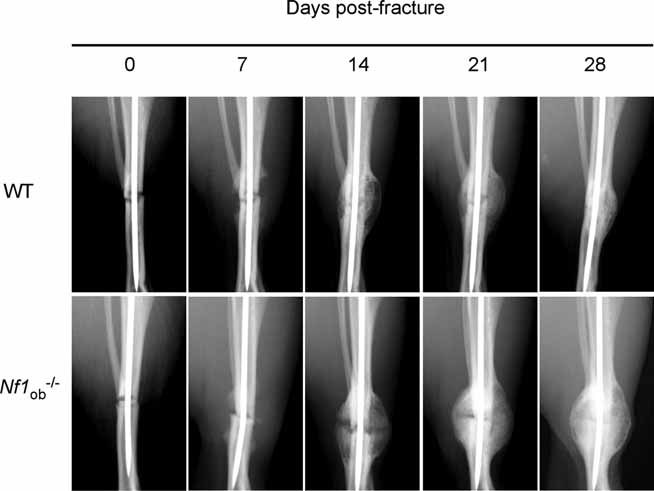
Lack of *Nf1* specifically in osteoblasts delays bone healing. Callus longitudinal X-ray analyses in WT and 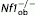 mice. Callus volume clearly decreased in the late stages of bone healing (days 21 to 28) in WT but not in 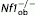 mice.

**Fig. 2:**
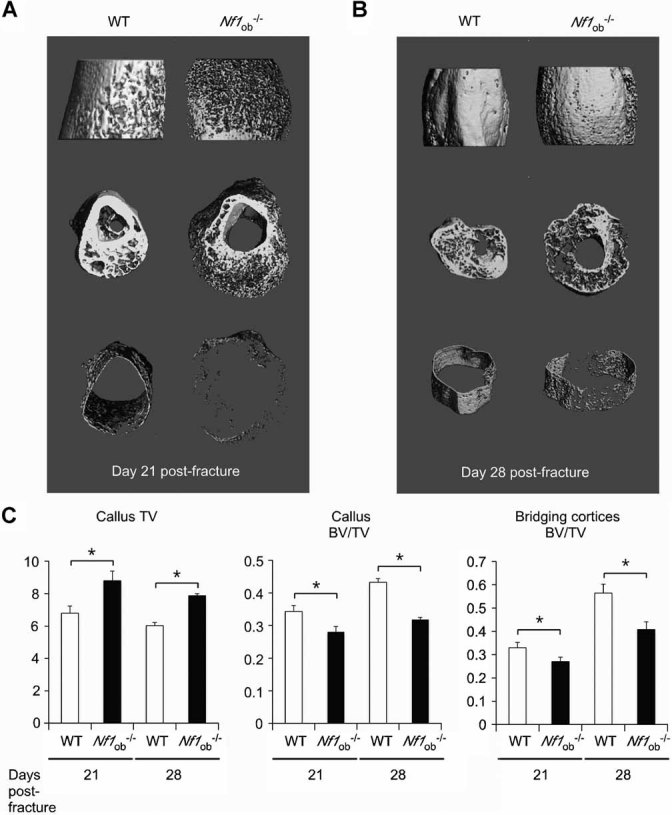
Callus trabecular and cortical volumetric parameters are affected by lack of *Nf1* in osteoblasts. (*A*, *B*) 3D µCT representative images from WT and 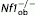 calluses on days 21 (*A*) and 28 (*B*) after fracture. Callus size was increased in mutant mice, whereas the amount of callus and bridging cortices calcified bone was decreased. Side view (*top panel*), cross view (*middle panel*), and bridging periosteum shell (*bottom panel*). (*C*) 3D µCT quantification of callus tissue volume (TV), callus bone volume over total volume (BV/TV), and bridging cortices BV/TV in WT and 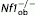 mice 21 and 28 days after fracture (*p < .05, *n* = 10 to 12 mice/group).

Partly calcified calluses were visible on X-ray 14 days after fracture, but no size or bridging difference was observed between genotypes (Fig. 1). Twenty-one days after fracture, the callus from WT mice was calcified and mostly bridged, as visible on µCT and X-ray images; in contrast, the callus from 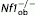 mice appeared larger (Figs. 1 and 2*A*), as quantified by a 29% increase in calcified tissue volume by 3D µCT (Fig. 2*A, C*). This increase in callus volume was accompanied by an 18% decrease in both callus BV/TV and callus bridging cortices BV/TV (Fig. 2*C*), suggesting abnormal bridging and potential poor mechanical properties. Twenty-eight days after fracture, callus volume decreased and callus BV/TV increased in both WT and 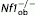 mice compared with the 21-day postfracture time point, indicating that the calluses in both genotypes were under active remodeling (Fig. 2*B, C*). However, the difference between WT and 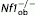 calluses further amplified to reach a 27% decrease in callus bridging cortices BV/TV and a 26% decrease in callus BV/TV in 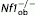 compared with WT calluses. In addition, both callus bridging cortices BMD (877.7 ± 69.0 versus 799.3 ± 94.4, *p* = .018) and callus BMD (1052.4 ± 53.1 versus 1000.8 ± 56.3, *p* = .019) were significantly decreased in mutant mice. Despite the poor mineral and structural quality of the newly formed calluses, union occurred in the mutant mice. These findings indicate that lack of *Nf1* specifically in osteoblasts delays postfracture bone healing.

### Lack of *Nf1* in osteoblasts decreases callus biomechanical properties

**Fig. 3:**
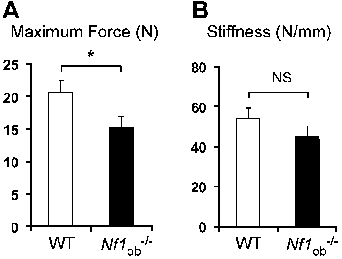
Lack of *Nf1* in osteoblasts weakens callus mechanical properties. (*A*) Callus strength (maximum force) as measured by three-point bending 28 days after fracture was significantly reduced in 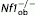 calluses. (*B*) Callus stiffness was reduced in 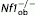 mice, but the difference between mutant and WT controls did not reach significance (NS) (*p < .05, *n* = 10 to 12 mice/group).

The significant higher callus volume and decreased callus and callus bridging cortices BMD and BV/TV observed in 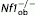 mice 28 days after fracture indicated that fracture healing was delayed when *Nf1* function was lost in mature osteoblasts. Most important, from a clinical point of view, these findings also suggested that calluses from 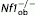 mice could be mechanically less robust than WT calluses. We thus asked whether callus stiffness and maximum force to failure, measured by three-point bending, were different between WT and 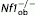 mice at a time point when calluses in WT mice were well bridged, calcified, and under active remodeling (day 28 after fracture). As shown in Fig. 3*A, B*, 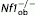 calluses displayed a significant 26% reduction in maximum force endured compared with WT littermate calluses and a 16% reduction in callus stiffness. These results thus indicate that *Nf1* in mature osteoblasts is required for optimal callus mechanical properties.

### Extensive osteoid surfaces and impaired osteoclast function may prevent proper callus remodeling in 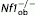 mice

**Fig. 4:**
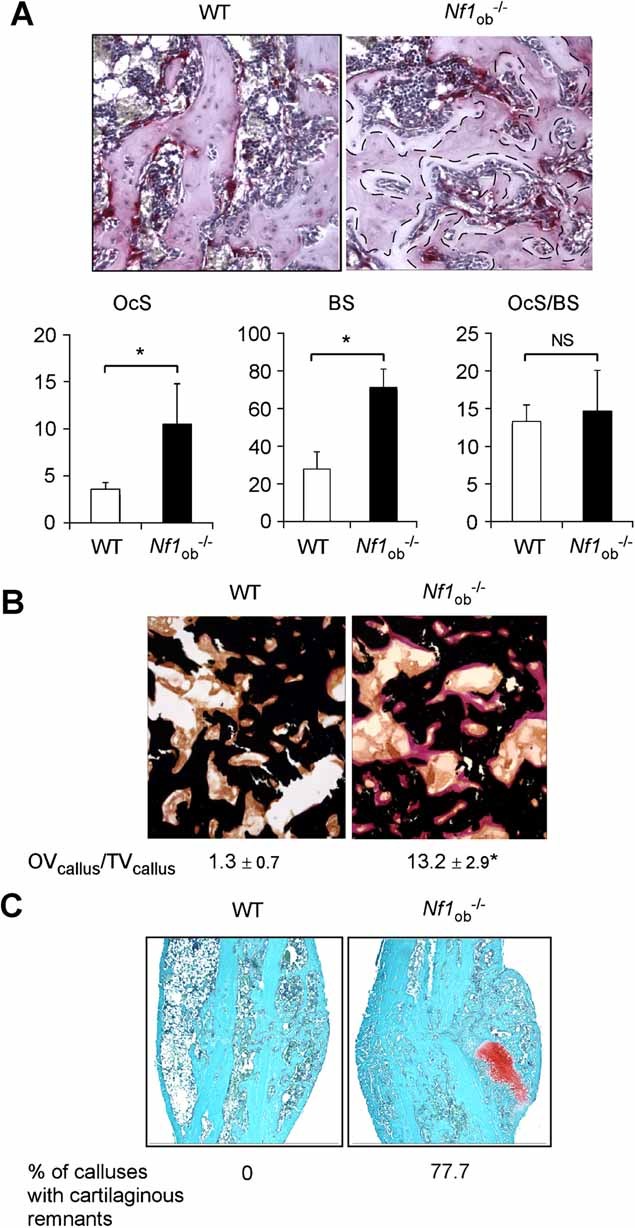
Increased osteoclast surface, osteoid, and number of cartilaginous remnants in 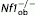 calluses. (*A*) TRAP^+^ osteoclast surface (OcS) and bone surface (BS) were significantly increased in 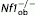 calluses 28 days after fracture compared with WT calluses (TRAP-stained and hematoxylin-counterstained). Osteoclast-free osteoid surfaces are underlined with a doted line. (*B*) Osteoid volume over bone volume (OV_callus_/BV_callus_) was significantly increased in 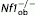 calluses compared with WT calluses (von Kossa/van Gieson staining) 28 days after fracture. (*C*) The number of cartilaginous remnants was significantly increased in 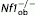 calluses compared with WT calluses 21 days after fracture (representative image of section stained with safranin-O and counterstained with methyl green) (*p < .05, *n* = 7 to 12 mice/group).

**Fig. 5:**
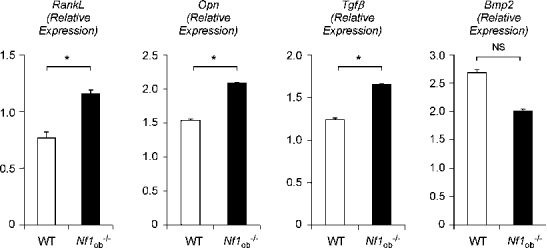
Gene expression changes induced by *Nf1* loss of function in osteoblasts. (*A*) *Rankl*, *Opn*, and *Tgfβ* but not *Bmp2* mRNA expressions were significantly increased 14 days after fracture in 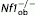 calluses compared with WT calluses. Gene expression was measured by quantitative TaqMan qRT-PCR and normalized by the expression of 18S rRNA (*p < .05, *n* = 3 to 4 calluses/group).

We further investigated the potential mechanism(s) whereby lack of *Nf1* in mature osteoblasts delays fracture healing by histomorphometric and gene expression studies. First, we observed that osteoclast-covered surfaces were significantly increased 28 days after fracture in mutant calluses (Fig. 4*A*), in agreement with an increase in *Rankl* expression measured in 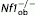 calluses (Fig. 5). Second, the presence of a thick osteoid matrix, visualized by von Kossa/van Gieson staining of undecalcified sections (Fig. 4*B*), was observed in mutant calluses. This significant increase in callus OV/TV was accompanied by increased expression of *Tgfβ* and *Opn*, two genes known to inhibit mineralization,(26–29) whereas *Bmp2* expression was not significantly affected (Fig. 5). On decalcified and hematoxylin-counterstained sections, this osteoid matrix (*pale purple staining, dotted lines*) was covered by osteoblasts and free of mature TRACP^+^ osteoclasts, restricting these latter resorbing cells to limited calcified (*darker purple staining*) bone surfaces (Fig. 4*A*). These observations suggested that callus remodeling by osteoclasts in 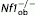 calluses is impaired by the accumulation of nonmineralized matrix. The existence of such a remodeling defect was supported by the presence of safranin O–stained cartilaginous matrix remnants in calluses from 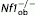 mice 21 days after fracture, a time point at which all cartilaginous elements have been resorbed in WT calluses (Fig. 4*C*).

### Lovastatins decreases ERK constitutive activation in *Nf1*^−/−^ osteoblasts in vitro

**Fig. 6:**
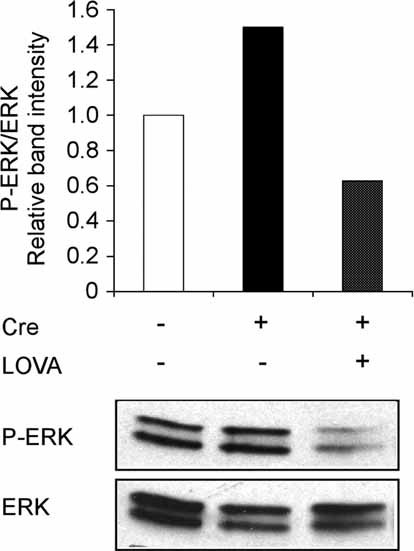
Lovastatin corrects ERK1/2 activation in *Nf1*^−/−^ osteoblasts. In vitro *Nf1* recombination by cre-adenovirus (+) (cre)-infection of *Nf1*^flox/flox^ osteoblasts increased ERK1/2 phosphorylation compared with mock (−) adenovirus-infection (*bottom panel*, representative blot, *n* = 3). Lovastatin (LOVA) treatment blunted this effect in cre-adenovirus (cre)-infected *Nf1*^flox/flox^ osteoblasts. The ratio P-ERK:ERK is quantified in the top panel.

RAS constitutive activation is a hallmark of *NF1*^−/−^ cells and causes most of the cellular defects related to NF1. Our previous studies(21) and others(16,22) demonstrated that lack of *Nf1* in osteoblasts leads to constitutive activation of RAS and of the downstream kinase ERK1/2. Based on these findings, we asked whether the inhibitory effect of lovatatin on HMG-CoA reductase and RAS prenylation and function(30,31) could decrease constitutive activation of ERK1/2 in *Nf1*^−/−^ osteoblasts. *Nf1*^flox/flox^ calvaria osteoblasts were isolated by collagenase digestion and were infected with cre-recombinase adrenoviruses to recombine *Nf1* in vitro 12 hours prior to Western blot analysis. Following cre-recombinase adrenovirus infection, ERK1/2 phosphorylation status was increased compared with GFP mock-transfected controls, indicating effective adenovirus-mediated recombination of the *Nf1*^flox/flox^ allele and activation of ERK1/2 signaling (Fig. 6). Lovastatin treatment (20 µM for 12 hours) consistently decreased ERK1/2 activation in *Nf1*^−/−^ osteoblasts compared with vehicle-treated controls. This in vitro proof-of-concept result suggested that lovastatin may be able to correct the dysfunctions of *Nf1*^−/−^ bone cells and improve bone healing when given to 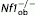 mice in vivo.

### Lovastatin microparticle treatment improves bone healing and mechanical properties in 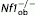 mice

**Fig. 7:**
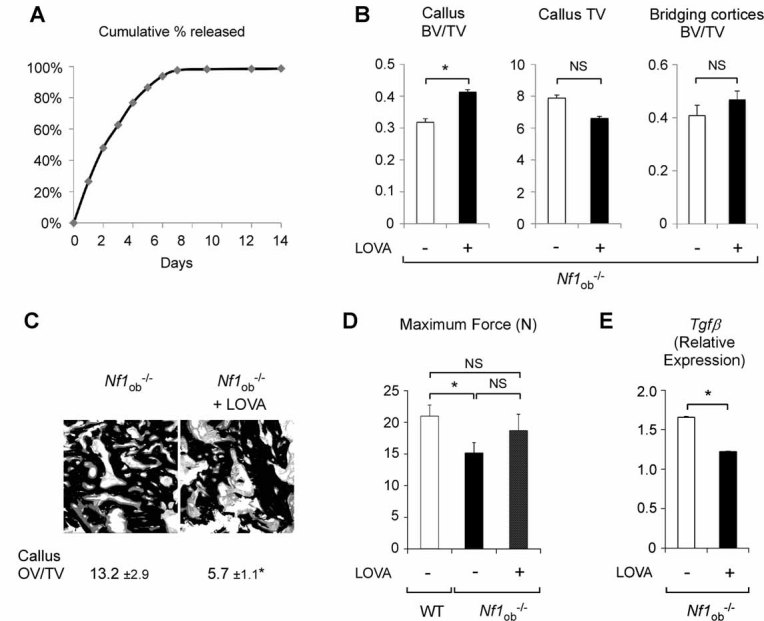
Lovastatin improves bone healing in 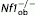 mice. (*A*) Lovastatin release profile. Cumulative release reached 100% within 8 days. (*B*) 3D µCT quantification of callus tissue volume (TV), callus bone volume over total volume (BV/TV), and callus bridging cortices BV/TV 28 days after fracture in 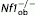 mice treated with lovastatin (LOVA) or control microparticles. (*C*) Decreased callus osteoid volume over total volume (OV_callus_/TV_callus_) in 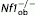 mice treated by lovastatin. (D) Lovastatin increased maximum force in 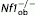 mice compared with control-treated 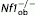 mice to an extent that this mechanical property was no longer different between WT control-treated mice and lovastatin-treated 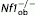 mice (*p < .05, *n* = 10 to 12 mice/group). (*E*) *Tgfβ* mRNA expression was decreased significantly by lovastatin 14 days after fracture in 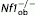 calluses. Gene expression was normalized by the expression of 18S rRNA (*p < .05, *n* = 3 to 4 per group).

High doses of statins given orally have a positive effect on bone mass in rodents,(32,33) but epidemiologic studies did not reveal a clear beneficial effect of statins on BMD or fracture risk at doses used to lower cholesterolemia(34–38) owing to the inability of the drug to reach bone cells as a consequence of first-pass metabolism in the liver. Importantly, delivery of low doses of statins in a way that bypasses liver metabolism improves bone mass and healing in rats.(39–41) Based on this information and on the in vitro proof of concept for lovastatin use described above (Fig. 6), we tested whether local and controlled delivery of low-dose lovastatin could improve the bone-healing defects observed in 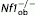 mice. Immediately following fracture, 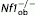 mice were treated with a single injection of control or lovastatin microparticles, which continuously release lovastatin for a period of 10 days (Fig. 7*A*) directly at the fracture site. Callus structural, cellular, and mechanical parameters were assessed 28 days after fracture in control and lovastatin microparticle–treated animals. Callus BV/TV was significantly higher (+30%) in lovastatin-treated 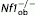 mice compared with vehicle-treated 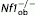 mice (Fig. 7*B*). Callus BMD also was significantly increased in lovastatin-treated compared with vehicle-treated 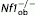 mice (1000.7 ± 56.3 versus 1068.2 ± 64.9, *p* = .01). Lovastatin caused a trend toward a reduction in callus volume and an increase of callus bridging cortices BV/TV in 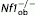 mice (16% and 15%, respectively; Fig. 7*B*). In addition to its effect on callus BV/TV and BMD, lovastatin reduced the number of cartilaginous remnants in 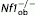 mice. Only 3 of 7 calluses contained cartilaginous remnants in lovastatin-treated 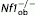 mice compared with 7 of 9 in vehicle-treated 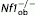 mice. Lovastatin also significantly reduced OV/TV in mutant calluses (Fig. 7*C*). From a mechanical point of view, callus strength in 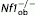 mice increased by 23% on lovastatin treatment (Fig. 7*D*) to an extent that made it no longer different from WT calluses. Lastly, lovastatin treatment significantly reduced *TGFβ* expression but did not affect *Rankl* and *Opn* expression 14 days after fracture in mutant calluses (Fig. 7*E* and data not shown). Together these results support a beneficial effect of low-dose local lovastatin delivery on several bone abnormalities identified in 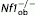 calluses and suggest that alternative regimens differing in dose, release profile, and time of injection may have the potential to further improve healing and mechanical properties in this NF1 model or others.

## Discussion

In this study we have used a genetic model of conditional *Nf1* inactivation in osteoblasts to demonstrate, first, that *Nf1* in osteoblasts is required for proper callus maturation and remodeling in vivo and, second, that attenuating ERK constitutive activation in *Nf1*^−/−^ osteoblasts by locally delivered lovastatin can ameliorate bone-healing and callus mechanical properties in 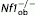 mice. Our results thus suggest that local release of lovastatin could improve bone healing in NF1 patients and support the choice of low-dose lovastatin microparticle strategy for future preclinical trials.

Previous studies using mice lacking one or two functional *Nf1* alleles in specific bone cell types provided experimental evidence supporting the notion that the NF1 skeletal defects are, at least in part, caused by a bone cell autonomous defect rather than a systemic effect.(16,21,22) These studies revealed the critical role of *NF1* in osteoblast biology and demonstrated that *NF1* is required for the differentiation and function of osteoblasts. However, none of these mouse models spontaneously sustained bone fracture and bone nonunion. This can be due to inherent bone structure differences between species, lower bone mechanical loading in mice compared with human, or most likely, the genetic manipulations used to generate these models, which do not fully recapitulate the hormonal (ie, low vitamin D and high parathyroid hormone) and cytokine/cellular context (such as the presence of a *NF1*^+/−^ stroma) of the human disease. Nevertheless, despite their phenotypic differences with NF1 patients, such mouse models can be useful to understand the role of *NF1* in specific bone cell populations and during specific time windows of the healing process. Using 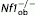 mice, we specifically addressed the role of *Nf1* in mature osteoblasts independent of its role in osteoprogenitor and osteoclast differentiation based on the specificity of the 2.3-kb mouse *type I collagen* promoter. Lack of *Nf1* in osteoblasts during bone healing caused a reduction in callus BV/TV and BMD and defective hard callus remodeling, eventually leading to an increase in callus size and reduced mechanical properties. The existence of an extensive nonmineralized matrix and the persistence of cartilaginous remnants, despite an overall increase in osteoclast surfaces, accompanied these structural phenotypes. Because *Nf1* is recombined specifically in mature osteoblasts in this model, it is unlikely that the presence of cartilage remnants observed at a late stage of healing in mutant calluses is due to a direct (cell autonomous) impairment of chondrocyte apoptosis or osteoclast activity. These results led us to speculate that *Nf1*^−/−^ osteoblasts may provide osteoclast and/or chondrocyte signals that impair their function or apoptosis, respectively. An alternative simple hypothesis is that the defect of callus maturation observed in 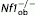 mice is caused by the formation of an abnormal bone matrix rich in osteoid that sterically impairs remodeling of the callus because of limited osteoclast access to cartilaginous and calcified bone surfaces. This hypothesis is supported by the observation that genetic or pharmacologic blockade of osteoclast activity in rodents enhances callus size.(42–45) Despite this size similarity, a major difference between these models and 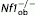 mice, however, is that callus structural and mechanical properties of rodents with impaired osteoclast function are increased, whereas these parameters are decreased in 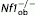 calluses. These observations strongly suggest that the bone laid down by *Nf1*^−/−^ osteoblasts has defective intrinsic properties leading to reduced callus mechanical strength in addition to increased callus size.

Several molecular defects could explain the increased amount of osteoid in 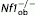 calluses. The expression of *type I collagen* and *Tnap*, two genes necessary and sufficient to induce bone ECM mineralization in mice,(46) was normal in 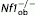 mice. In addition, hypophosphatemia was not observed in 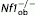 mice,(21) excluding the contribution of fibroblast growth factor 23 (FGF-23) and Phex in this mineralization phenotype. However, *Nf1*^−/−^ osteoblasts were found to secrete higher amount of type I collagen than WT osteoblasts, possibly overriding their mineralization potential and leading to the mineralization lag time reported earlier.(21) The increase in *Opn* and *TGFβ* expression observed in 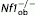 calluses might contribute directly to the severe osteoidosis observed in these calluses as well because both genes have been proposed to inhibit the bone mineralization process(26–29); however, no experimental evidence exists at this point to determine if these two factors significantly contribute to this phenotype.

Despite delayed bone healing, decreased biomechanical strength, increased osteoclast number, and the presence of large osteoid surfaces, which are features shared with NF1 patients,(47,48) the calluses of 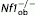 mice appear bridged (although incompletely), which is distinct from the clinical observations from NF1 patients, who display bone nonunion (pseudoarthrosis).(2,5,14,49,50) This difference can occur for a number of reasons. On the one hand, 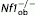 mice are genetically different from NF1 patients: 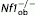 mice lack both copies of *Nf1* in all mature osteoblasts, but other cells of the bone marrow environment (including osteoprogenitors) and body are normal (ie, possess both functional *Nf1* alleles); NF1 patients, on the other hand, have one functional *NF1* allele in every cell, whose function may be lost in restricted bone cell subpopulations. It is presently unclear what exact bone cell type and what proportion of such cells are defective in NF1 pseudoarthrosis lesions and whether the haploinsufficient stroma contributes to these defects. Based on fact that *Nf1*^+/−^ and *Nf1*^−/−^ osteoprogenitor cells show defective differentiation and that *Nf1*^+/−^ mice display a delay in bone healing,(16,22,51) it seems reasonable to hypothesize that *Nf1* loss of function in osteochondroprogenitors, combined with the presence of a *Nf1*^+/−^ stroma, could trigger a more pronounced healing-defect phenotype and possibly bone nonunion owing to the combination of defective osteoblast progenitor differentiation and abnormal mature osteoblast function. The delayed bone healing observed following cortical bone lesions in the *Nf1*^Prx^ mouse model supports this hypothesis.(52)

A hallmark of *NF1*-deficient cells is constitutive activation of RAS and ERK signaling. Therefore, pharmacologic interventions aimed at attenuating this specific molecular defect have become a logical strategy to prevent or correct NF1-related bone abnormalities. Lovastatin has generated a great deal of interest in the NF1 field for several reasons. The first is that this drug inhibits the mevalonate pathway and indirectly the prenylation of small G protein–like RAS, thereby inhibiting their membrane localization and function. Therefore, such a drug had the potential to correct NF1-specific molecular defects. Supporting this rationale, oral high-dose lovastatin has shown encouraging effects at reducing learning deficits in *Nf1*^+/−^ mice.(53) Second, lovastatin has been shown to have bone anabolic properties and to promote bone healing in rats, provided that it is given in the form of local delivery to avoid liver metabolism.(40) The results of our study indicate that local lovastatin delivery may be particularly well suited for the treatment of NF1 bone-healing abnormalities. At a molecular level, lovastatin could rescue the increase in callus *TGFβ* expression but not the increase in *Rankl* and *Opn* expression caused by lack of *Nf1* in osteoblasts. The absence of effect of lovastatin on *Rankl* expression is not surprising because we have shown previously that lack of *Nf1* increases *Rankl* expression via PKA activation and phosphorylation of ATF4 at serine 254,(21) which is independent from the RAS/ERK/RSK2/ATF4 serine 251 pathway targeted by lovastatin. On the other hand, the effect of lovastatin on *TGFβ* expression suggests that *TGFβ* expression may be regulated by *Nf1* via the RAS/ERK pathway. The beneficial effect of lovastatin on OV/TV and callus BV/TV in mutant mice strongly suggests that it acts, as expected, by reducing the activation of the RAS/ERK pathway in *Nf1*^−/−^ bone-forming cells. Most important, from a clinical point of view, these molecular and structural beneficial effects were followed by improvements in callus mechanical properties, which thus confirm that targeting the specific defects caused by loss of *Nf1* function could be an efficient strategy to improve bone healing in NF1 patients. A clear advantage of such a pharmacologic strategy is that the slow and local release of the drug provides a sustained and efficient local correction of the molecular defects specific to *Nf1*^−/−^ cells regardless of their exact nature. Clinically, such an approach also has the advantage of a single injection at the time of fracture correction, which can be combined with additional pharmacologic and surgical methods to stabilize and promote healing. It also avoids the use of high doses of lovastatin in children and possible secondary effects. Future studies using different doses and release profiles should allow us to further improve the positive effects observed in this study and to narrow down the still wide gap between mouse preclinical models and NF1 clinical trials.
